# Destruction of Forests

**Published:** 1881-05

**Authors:** T. L. Lewis


### DESTRUCTION OF FORESTS.

The indifference of our people to forestry is criminal. It has been estimated that there are but six states east of the Rocky Mountains which have a surplus of timber; yet, nearly all are denuding their lands and shipping wood, ties and lumber. Strike a line through Minnesota, Iowa, Kansas, and Texas; or take longitude 80° west of Washington, and the eastern side contains all the timber, while the western is almost one boundless desert of treeless prairie. California is far above an average, yet only about one-twentieth part is timbered. This western world of prairie is now teeming with active life, and must soon be the home of millions, with their shops, factories, railroads, etc., etc. It becomes then a practical question—will the supply meet the demands under our present reckless system of the wanton destruction of our forest? It is an impossibility unless the timbered states legislate wisely on this matter, and the prairie states follow the example of Kansas in the cultivation of forests. Let us look at a few facts.

It has been estimated that 30,000,000 of our people use wood as fuel, consuming 100,000,000 cords annually. We have about 90,000 miles of railroads consuming 400,000 acres of timber every year. Steamboats, factories, brick yards, etc., consume annually about 35,000,000 cords. And more than 70,000 factories of wood articles also consume about $410,000,000 of timber every year. The total forest cutting annually is estimated at nearly one thousand millions of dollars. Again we ask, at this enormous and wholesale rate of forest destruction, can the supply meet the demands of the rising west and still provide for the wants of the east, unless our forests are protected by law?—*T. L. Lewis, Kansas City Review.*

